# Retrospective Analyses of Potential Risk Factors for Posterior Capsule Opacification after Cataract Surgery

**DOI:** 10.1155/2018/9089285

**Published:** 2018-08-05

**Authors:** Shuang Wu, Nianting Tong, Lin Pan, Xiaohui Jiang, Yanan Li, MeiLing Guo, Hehuan Li

**Affiliations:** ^1^Qingdao Municipal Hospital Affiliated to Qingdao University, No. 5 Donghaizhong Road, Shinan District, Qingdao, Shandong, China; ^2^Department of Ophthalmology, Qingdao Municipal Hospital, No. 5 Donghaizhong Road, Shinan District, Qingdao, Shandong, China; ^3^Dalian Medical University, No. 9 Lushunnan Road, Dalian, Liaoning, China

## Abstract

**Purpose:**

To evaluate the potential risk factors of posterior capsule opacification (PCO) after cataract surgery.

**Methods:**

Data on PCO patients diagnosed from September 2015 to May 2017 were obtained from the Department of Ophthalmology at Qingdao Municipal Hospital, Qingdao, China. The factors associated with PCO were assessed using Pearson's *χ*^2^ test for univariate analyses and logistic regression for multivariate analyses.

**Results:**

Eyes (652) from 550 patients were enrolled in this study. All patients were diagnosed with PCO/non-PCO and had <3 years of follow-up after surgery. The numbers of PCO and non-PCO were 108 eyes and 544 eyes, respectively. Statistically significant associations with PCO were found for age at the time of surgery (*χ*^2^ = 78.504; *p* < 0.001), diabetes (*χ*^2^ = 4.829; *p*=0.028), immune diseases (*χ*^2^ = 4.234; *p*=0.004), high myopia (*χ*^2^ = 5.753; *p*=0.016), lens nucleus hardness (*χ*^2^ = 11.046; *p*=0.026), surgery type (*χ*^2^ = 11.354; *p*=0.001), a history of vitrectomy (*χ*^2^ = 4.212; *p*=0.004), ocular inflammation (*χ*^2^ = 6.01; *p*=0.009), and the intraocular lens (IOL) type (*χ*^2^ = 8.696; *p*=0.003). Multivariable data analyses using logistic regression analyses of the variables showed that age at the time of surgery <60 years, diabetes, lens nucleus hardness of III–V, extracapsular cataract extraction (ECCE), postvitrectomy, and hydrophilic IOLs were significant independent risk factors associated with PCO.

**Conclusions:**

Age <60 years, diabetes, lens nucleus hardness of III–V, ECCE, postvitrectomy, and a hydrophilic IOL were significantly associated with the formation of PCO. Estimation of the incidence of and risk factors for PCO should help in patients counseling and in the design of treatment protocols to reduce or prevent its development.

## 1. Introduction

Cataract is the most common cause of blindness, and cataract surgery is the only cure method performed. Phacoemulsification and extracapsular cataract extraction (ECCE) are the most common types of surgery to treat cataracts. Posterior capsule opacification (PCO) is one of the most common complications after surgery. Decreased visual acuity induced by PCO is reported to occur in 20%–40% of patients 2–5 years after surgery [1]. PCO in the central visual axis is usually treated with neodymium : YAG (Nd : YAG) laser capsulotomy. The cumulative incidence of Nd : YAG capsulotomy were 10.6%, 14.8%, 21.2%, and 28.6% in the patients after 1, 2, 3, and 4 years [2]. Because this treatment risks complications in other structures of the eye, the need for PCO prevention becomes increasingly important. During the past decades, various forms of prevention have been used, including general measures during surgery (e.g., surgical techniques, intraocular lens (IOL) materials, and designs), pharmacological prevention, and the prevention of PCO by interfering with the biological processes of epithelial-to-mesenchymal transition (EMT) in lens epithelial cells (LECs) [3]. This is a lack of quantified information on these processes, and the only effective method is mechanical prevention of PCO formation. A low incidence of PCO after IOL implantation is therefore a key objective of most cataract surgeries.

Many previous cohort or randomized controlled studies that have assessed PCO after cataract surgery had small sample sizes or had postoperative outcomes involving a few, mainly senior, surgeons. These studies did not adjust for different surgeons or different surgical experiences. It remains unclear whether such findings are applicable to other settings, such as that of large sample sizes in a public tertiary hospital where a large number of cataract procedures are performed using different ophthalmologic surgeons.

In this study, we assessed the incidence of PCO within 3 years after cataract surgery, to identify preoperative and surgical factors associated with PCO in the study population. Factors such as the general condition of the patient, ocular conditions, surgical techniques, and IOL types were considered. Our results should help with evaluations of the long-term prognosis of cataract surgery, and provide early diagnosis and treatment options for high-risk patients.

## 2. Materials and Methods

In this retrospective case-control study, the incidence and risk factors of PCO were evaluated in patients who underwent phacoemulsification or ECCE, with a follow-up time <3 years. The hospital's Institutional Review Board approved the study protocol. Informed consent was obtained from all patients or their guardians before the study, and the study was performed at Qingdao Municipal Hospital, Qingdao, China. The medical records of patients with PCO and who visited the outpatient department of the Department of Ophthalmology between September 2015 and May 2017 were retrospectively reviewed. A group of patients without PCO was identified using a cumulative sampling strategy. The patients complied with the criteria for the diagnosis of PCO and were selected at a 1 : 5 case : control ratio with the non-PCO group. Surgery was uncomplicated in all cases. Both groups of patients received antibiotic and glucocorticoid eye drops during the follow-up period within 4 weeks after the surgery according to the intraocular inflammation. There was no difference in postoperative care between the two groups of patients.

**Figure 1:**
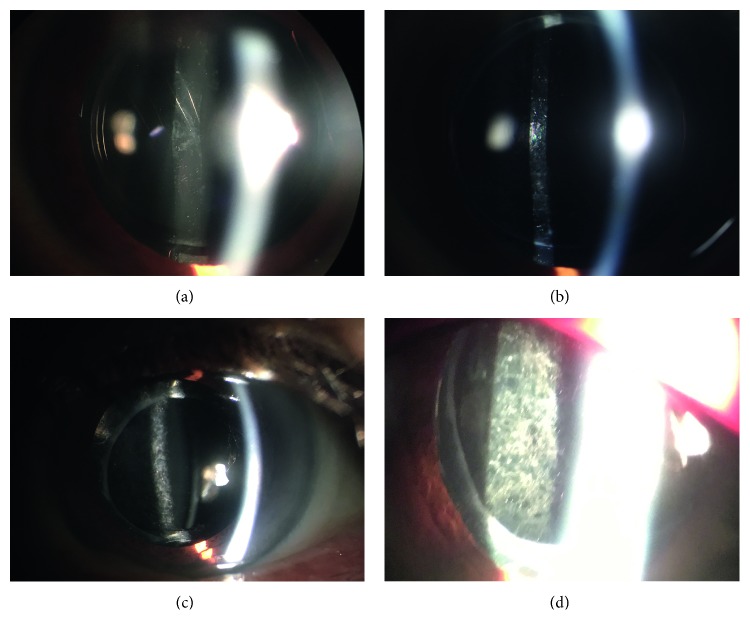
PCO grades by slit-lamp examination and a Volk 90D lens in this study: (a) Grade 0. (b) Grade 1. (c) Grade 2. (d) Grade 3.

The PCO and lens nucleus hardness classification was evaluated by the same physician (XHJ). Visualisation of the posterior pole was assessed by examining the optic disc and macula using a Volk 90D lens. Visualisation of the optic disc was subjectively graded according to the following scale: 0—clear view of optic disc margin, blood vessels at the optic disc, and nerve fibre layer (NFL examined using the red-free filter); 1—clear view of optic disc margin, but disc blood vessels and/or nerve fibre layer are not clearly seen; 2—optic disc margin, as well as disc blood vessels, and nerve fibre layer are not clearly seen. Visualisation of the macula was subjectively graded according to the following scale: 0—clear view of foveal reflex, perifoveal blood vessels, and nerve fibre layer; 1—diminished foveal reflex, but clear view of perifoveal blood vessels and nerve fibre layer; 2—blurred foveal reflex, perifoveal blood vessels, and/or nerve fibre layer. The totals for the visualisations of the optic disc and the macula were combined to produce a total posterior pole visualisation score (PolVS), ranging from 0 to 4 (Figures 1(a)–1(d)) in order of decreasing visualisation [4]. The degree of lens nucleus hardness was classified according to the Emery–Little classification (grade I–V). Patients were excluded if they underwent cataract surgery more than 3 years prior to the study, had a history of ocular trauma, had a history of glaucoma filtration surgery, had the primary aphakia after cataract extraction, had a history of repeated vitrectomy after cataract surgery, had the intraoperative posterior capsule rupture, had a history of extracapsular fixation of the IOL, were unable to cooperate with the inspector, or had incomplete clinical information. All patients presented with decreased visual acuity which was attributed to PCO during study period in our clinic. Nd : YAG laser capsulotomy was performed if it was clinically indicated by a decrease in visual acuity of two lines at least since previous examination or in the presence of a clinically opaque capsule. Nd : YAG laser capsulotomy was eventually performed in all 92 eyes.

The data collected included age at the time of surgery, sex, cataract duration, surgery type, IOL materials, ocular inflammation, lens nucleus hardness, family history of cataracts, and personal history of smoking, history of drinking, diabetes, hypertension, immune diseases, high myopia, and vitrectomy. Follow-up examinations included visual acuity measurement, intraocular pressure measurement using Goldman applanation tonometry, evaluation of IOL centration, and dilated fundus examination. The data analyses were performed using SPSS statistical software for Windows, version 19.0 (SPSS, Chicago, IL, USA). Pearson's *χ*^2^ test was used to determine if there were statistically significant differences in PCO incidence for each factor. Then the factors that were statistically significant at a univariate level were entered into a stepwise multiple logistic regression model to identify independent risk factors affecting PCO. The 95% confidence interval (CI) and odds ratio (OR) were calculated. A value of *p* < 0.05 was considered statistically significant.

## 3. Results

This retrospective study reviewed the records of 108 eyes of 92 PCO patients in PCO group, including 52 females and 40 males, with a mean age of 65.47 ± 15.32 years. A total of 544 eyes of 458 patients, including 231 females and 227 males, with a mean age of 64.77 ± 10.12 years, were evaluated in the non-PCO group. There was no difference in the mean age between the PCO group and non-PCO group (*p*=0.041). The period from surgery to the follow-up visit was on average 26.04 ± 8.14 months in PCO group and non-PCO group at 25.27 ± 7.67 months (*p*=0.65). All comparative statistics are shown in Table 1. Patients with various PCO grades are listed in Table 2.

Table 3 shows the associations between PCO and clinical characteristics. No statistically significant differences were found between the two groups (PCO versus non-PCO) for sex, cataract duration, and history of smoking, history of drinking, hypertension, and cataracts. The *χ*^2^ shown for clinical characteristics associated with PCO was based on univariate analyses. Variables with statistically significant differences were age at time of surgery, lens nucleus hardness, surgery type, ocular inflammation, IOL materials, and a history of diabetes, immune diseases, high myopia, and vitrectomy (Table 3). Variables with *p* < 0.05 were included in multiple logistic regression analyses (Table 4). Age at time of surgery <60 years (OR, 0.149; 95% CI, 0.092–0.241; *p*=0.000), diabetes (OR, 1.825; 95% CI, 1.042–3.197; *p*=0.035), a lens nucleus hardness of III–V (OR, 0.508; 95% CI, 0.304–0.847; *p*=0.009), ECCE (OR, 2.563; 95% CI, 1.242–5.290; *p*=0.011), vitreous loss (OR, 1.905; 95% CI, 1.046–3.471; *p*=0.035), and a hydrophilic IOL (OR, 1.672; 95% CI, 1.043–2.682; *p*=0.033) were significant independent factors associated with PCO.

## 4. Discussion

PCO is caused by proliferation and migration of residual LECs, fibroblasts, macrophages, and iris-derived pigment cells on the posterior capsule. All of these processes are influenced by cytokines, growth factors, and extracellular matrix proteins. Clinically, there are two morphological types of PCO, including the fibrosis type and the pearl type. The fibrosis type is caused by the proliferation and migration of LECs, which undergo EMT, resulting in fibrous metaplasia and leading to significant visual loss because of folds and wrinkles in the posterior capsule. Pearl-type PCO is caused by LECs located at the equatorial lens region (lens bow), causing regeneration of crystallin-expressing lenticular fibers and forming Elschnig pearls and Soemmering rings, which are responsible for most cases of PCO-related visual losses. At present, the molecular mechanisms influencing leftover LECs behavior after cataract surgery are not completely known. Many other mechanisms may also affect the posterior capsule. Remnants of the lens may become trapped, absorb water, and appear fluffy white. Folds or tears leading to mechanical distortion of the bag may cause irregularities in posterior capsule transparency. Posterior inflammation may lead to deposits of proteins and white blood cells on the capsule, while surgical trauma may lead to deposition of red blood cells and pigmented cells [5]. A number of potentially significant factors in the development of PCO have been identified, including IOL materials, design and placement, surgical techniques, cortical clean-up, and concomitant pathologies.

Young age is a known risk factor for PCO, as shown by several previous studies and confirmed by our study. There are more LECs on the anterior capsules of young patients. A higher percentage of LECs retain strong cellular proliferative activity in young patients compared to older patients. In addition, the levels of hormones and cytokines in the aqueous humor are more suitable for LECs growth. Previous studies have reported that the rate of LECs growth is age-dependent, and the rate in young patients <40 years of age is three times faster than that in patients >60 years of age [6]. Furthermore, the nuclear hardness of the lens gradually increases with growth during aging, which may affect the choice of surgery.

Not even one surgical approach involving lens extraction is able to completely remove the residual LECs. It has recently been suggested that almost 100% elimination of residual LECs may be necessary to prevent LECs proliferation on the posterior capsule and the development of PCO [7]. A comparison of PCO formation using different surgical techniques in previous studies is therefore controversial. Previous studies have also found that phacoemulsification and cortical I/A lead to less residual LECs concentrations on the internal capsule surfaces than ECCE with manual nuclear expression [7]. In addition, Nishi and Nishi [8] reported that meticulous capsule vacuuming using ultrasound endocapsular cataract surgery significantly reduces the need for laser capsulotomy (in their study, from 10.8% to 3.7%). Furthermore, phacoemulsification reduces damage to the blood-aqueous barrier by requiring only a minor surgical incision compared to ECCE, particularly in patients with diabetes [9]. Phacoemulsification uses continuous curvilinear capsulorhexis to decrease the capsular space of proliferation and migration of residual LECs [10]. All of these factors are considered to increase the formation of PCO in ECCE compared to phacoemulsification. Our findings are similar to these previous clinical and basic studies. However, a different point was raised that cell survival and growth were dependent on the patient's general condition, rather than the surgical technique performed. Some other studies have also reported no significant differences between phacoemulsification and ECCE surgery in terms of the *in vitr*o percentage of PCO formation using a human capsular bag model [11]. In our study, the patients were followed for different periods of time, which may have affected the results (due to the use of updated surgical materials and equipment). However, this protocol provided a means of studying clinically important factors.

There have been many previous studies on the relationship between diabetes and the development of PCO. Several studies have reported significantly greater PCO formation in diabetic patients compared to nondiabetic patients, while others have reported no difference or a lower incidence of PCO in diabetic patients. The difference between the formation mechanism of PCO in patients with diabetes and nondiabetic patients is presently unclear. Praveen et al. [12] performed an observational case-control study to compare the development of PCO between eyes with and without diabetes after phacoemulsification and implantation of a single-piece hydrophobic acrylic IOL, with a 4-year follow-up. They reported a higher incidence of PCO in diabetic patients for up to 12 postoperative months. At the 4-year follow-up, there were no significant differences between the two groups. They also reported that the duration of diabetes increased the risk for PCO and the severity of retinopathy but did not influence the development of PCO. Hayashi et al. [13] reported similar findings at 36 months of follow-up. However, Elgohary and Dowler [2] suggested that diabetic patients had a lower long-term incidence and decreased risk for PCO at 4 years. Zaczek and Zetterstrom [14] reported similar findings at 2 years. A possible explanation for the lower PCO in diabetic patients may be the decreased density of LECs in the diabetic capsular bag [15] and/or the detrimental effects of accumulating intracellular sorbitol [16] and fructose, free radicals, and oxidative stress on their survival and proliferative capacities [17]. These effects may explain the decrease in the incidence of PCO in diabetic patients at longer follow-up periods. However, our study suggests that diabetic patients are at increased risk for developing PCO. Some studies have attributed this phenomenon to damage to the blood-aqueous barrier and increased inflammation in the aqueous chamber [18]. Several clinical and experimental studies have reported that the incidence of LECs proliferation increases in protein-rich environments [19], which may subsequently result in extensive PCO.

A limitation of the present study is that we did not record the stage of diabetic retinopathy or blood glucose levels, which may be correlated with the degree of PCO in diabetic patients. In previous studies, the observation time of the subjects was fixed for long-term follow-up after surgery, whereas the observation times of our patients varied within 3 years, which may partially explain these discrepancies. Although they appear to be related, it is likely that inflammation, LECs proliferation, and PCO formation are independent of each other.

In previous studies, the incidence of PCO was significantly higher after cataract surgery using vitrectomy than that after cataract surgery alone, particularly in diabetic patients [20]. We found that combined surgery with vitrectomy was also an independent risk factor for the progression of PCO, consistent with other reports. Most reports have concluded that combined surgery of eyes could result in severe postoperative inflammation, which presumably leads to more extensive PCO. Such studies have suggested that elevated levels of cytokines caused by postoperative inflammation accelerate LECs proliferation via autocrine and/or paracrine signaling. Furthermore, it has recently been reported that there is more extensive PCO formation using 20-gauge phacovitrectomy than when 23-gauge phacovitrectomy is used to lower postoperative inflammation [21]. In addition, posterior vitreous pressure, the hypoxic state, intraocular gas tamponade, and silicone oil tamponade are associated with LECs proliferation, migration, and transdifferentiation after combined surgery. Meanwhile, we cannot differentiate whether the formation of PCO influenced by the factors of other vitreo-retinal disease, for which combined surgery is necessary.

Previous studies have reported a high incidence of PCO and subsequent Nd : YAG laser posterior capsulotomy in highly myopic eyes. High myopia is pathologic and is associated with an increase in certain growth factors in the aqueous humor, which might influence the development of PCO. However, the degree of PCO and the incidence of Nd : YAG capsulotomy in myopia eyes may be relatively low [22]. Furthermore, Hayashi et al. [23] reported that a long axial length is not a risk factor for the formation of PCO. However, these results on the role of myopia in PCO might not be extrapolated from clinical studies that have defined myopia based on different standards, such as axial length, the power of the implanted IOL, and the preoperative refraction. In the present study, it was difficult to clearly correlate the development of PCO with the factors of age, refractive lens exchange, or solely myopia. Nonetheless, our results could help surgeons estimate the incidence of PCO in myopic eyes and make decisions on the timing of Nd : YAG to minimize the risk of PCO formation after cataract surgery.

In the present study, IOL materials were either hydrophobic or hydrophilic. Previous studies have reported that the incidence of PCO in patients implanted with hydrophilic lenses is higher than the in patients implanted with hydrophobic lenses [24]. However, some studies have reported that the incidence of PCO using hydrophilic lenses is less than in patients using hydrophobic lenses [25, 26]. In addition to the optic material of IOLs, there are many other IOL factors that might influence the formation of PCO, such as the overall length, optic diameter, optic edge, haptic design, haptic material, and incision size needed for implantation. In the study by Findl et al. [27], a sharp-edged optic inhibited lens epithelial cells growth and lowered the incidence of PCO and laser capsulotomy. Schriefl et al. [28] reported that microincision cataract surgery of IOLs has a higher PCO incidence than conventional IOLs. Previous studies of cadaveric eyes have reported that Acrysof® has a relatively low propensity to induce cell proliferation in the capsular bag. Therefore, our study has a significant limitation, because only IOL material factors were analyzed. However, our results still have some clinical significance, because the incidence of PCO was higher in hydrophilic than in hydrophobic IOLs. Because the hydrophilic acrylic lenses have a different water content, there is a greater probability of contacting the lens capsule, which is more suitable to the “no space no cells” theory [29].

Other factors are thought to contribute to the formation of PCO, including IOL decentration, capsulorhexis decentration, capsule tears, and insufficient zonules, but we did not analyze these factors. We only compared the outcomes of surgeries performed using different surgeons at different institutions, and the patients were followed up for different periods of time. Any differences that occurred in the incidences of PCO may have resulted from factors not included in this study. In addition, the independent variable was not comprehensive, which may have affected the results of the logistic regression model. In future studies, we need to collect more clinical records of cases to improve the independent variable and conduct multicenter logistic regression analyses on a larger sample using a prospective study design.

## Figures and Tables

**Table 1:** Patient demographics.

Parameter	Group		*χ* ^2^	*p* value
	Non-PCO	PCO		
Eye, *n* (patients)	544 (458)	108 (92)		
Age (y)	64.77 ± 10.12	65.47 ± 15.32	0.932	0.41
Laterality (OD/OS)	265/279	52/56	0.803	0.47
Period from surgery to the follow-up visit (m)	25.27 ± 7.67	26.04 ± 8.14	0.587	0.65

**Table 2:** Incidence and PCO grade among PCO and non-PCO patients.

	Incidence (%)	CDVA (logMAR) ± SD
Non-PCO		
Grade 0	544 (1)	0.06 ± 0.02

PCO		
Grade 1	46 (0.426)	0.27 ± 0.08
Grade 2	44 (0.407)	0.49 ± 0.13
Grade 3	18 (0.167)	0.74 ± 0.12

CDVA = corrected distance visual acuity; logMAR = logarithm of the minimum angle of resolution; SD = standard deviation.

**Table 3:** Univariate analyses of factors associated with PCO.

Variable	PCO (*n*=108)	Non-PCO (*n*=544)	*χ* ^2^	*p* value
Sex				
Male	46	276		
Female	62	68	2.39	0.122

Age at the time of surgery				
>60 years old	32	401		
<60 years old	76	143	78.504	<0.001

Cataract duration				
>3 years	79	390		
<3 years	29	154	0.064	0.8

History of smoking				
Yes	30	161		
No	78	383	0.144	0.705

History of drinking				
Yes	26	134		
No	82	410	0.015	0.902

History of hypertension				
Yes	52	287		
No	56	257	0.767	0.381

History of diabetes				
Yes	27	88		
No	81	456	4.829	0.028

History of immune diseases				
Yes	16	46		
No	92	498	4.234	0.04

High myopia				
Yes	13	31		
No	95	513	5.753	0.016

Family history of cataract				
Yes	33	175		
No	75	369	0.108	0.742

Lens nucleus hardness				
I grade	7	70		
II grade	22	145		
III grade	38	183		
IV grade	33	130		
V grade	8	16	11.046	0.026

Surgery type				
ECCE	18	37		
Phaco	90	507	11.354	0.001

History of vitrectomy				
Yes	23	74		
No	85	470	4.212	0.04

Ocular inflammation (aqueous cell)				
>15 cells	38	126		
<15 cells	70	418	6.01	0.009

IOL materials				
Hydrophilic	67	253		
Hydrophobic	41	291	8.696	0.003

**Table 4:** Significant risk factors for PCO based on variate logistic regression.

Variable	Regression coefficient	OR	95% CI		*p* value
Age at time of surgery					
>60 years old	Reference	0.149	0.092	0.241	0.000
<60 years old	−1.904				

History of diabetes					
No	Reference	1.825	1.042	3.197	0.035
Yes	0.602				

History of immune diseases					
No	Reference	1.128	0.471	2.700	0.787
Yes	0.120				

History of high myopia					
No	Reference	2.344	0.884	6.217	0.087
Yes	0.852				

The lens nucleus hardness					
I-II grade	Reference	0.508	0.304	0.847	0.009
III–V grade	−0.678				

Surgery type					
Phaco	Reference	2.563	1.242	5.290	0.011
ECCE	0.941				

History of vitrectomy					
No	Reference	1.905	1.046	3.471	0.035
Yes	0.645				

Ocular inflammation(aqueous cell)					
<15 cells	Reference	1.286	0.757	2.182	0.352
>15 cells	0.251				

IOL materials					
Hydrophilic	Reference	1.672	1.043	2.682	0.033
Hydrophobic	0.514				

## Data Availability

The data used to support the findings of this study are included within the article. No additional unpublished data are available.
